# Differential structure-function network coupling in the inattentive and combined types of attention deficit hyperactivity disorder

**DOI:** 10.1371/journal.pone.0260295

**Published:** 2021-12-01

**Authors:** Dongha Lee, Elizabeth Quattrocki Knight, Hyunjoo Song, Saebyul Lee, Chongwon Pae, Sol Yoo, Hae-Jeong Park

**Affiliations:** 1 Center for Systems and Translational Brain Sciences, Institute of Human Complexity and Systems Science, Yonsei University, Seoul, Republic of Korea; 2 Cognitive Science Research Group, Korea Brain Research Institute, Daegu, Republic of Korea; 3 Department of Psychiatry, Harvard Medical School, McLean Hospital, Belmont, Massachusetts, United States of America; 4 Department of Educational Psychology, Seoul Women’s University, Seoul, Republic of Korea; 5 Department of Nuclear Medicine, Department of Psychiatry, Yonsei University College of Medicine, Seoul, Republic of Korea; 6 Department of Cognitive Science, Yonsei University, Seoul, Republic of Korea; 7 Graduate School of Medical Science, Brain Korea 21 Project, Yonsei University College of Medicine, Seoul, Republic of Korea; Istituto Italiano di Tecnologia, ITALY

## Abstract

The heterogeneous presentation of inattentive and hyperactive-impulsive core symptoms in attention deficit hyperactivity disorder (ADHD) warrants further investigation into brain network connectivity as a basis for subtype divisions in this prevalent disorder. With diffusion and resting-state functional magnetic resonance imaging data from the Healthy Brain Network database, we analyzed both structural and functional network efficiency and structure-functional network (SC-FC) coupling at the default mode (DMN), executive control (ECN), and salience (SAN) intrinsic networks in 201 children diagnosed with the inattentive subtype (ADHD-I), the combined subtype (ADHD-C), and typically developing children (TDC) to characterize ADHD symptoms relative to TDC and to test differences between ADHD subtypes. Relative to TDC, children with ADHD had lower structural connectivity and network efficiency in the DMN, without significant group differences in functional networks. Children with ADHD-C had higher SC-FC coupling, a finding consistent with diminished cognitive flexibility, for all subnetworks compared to TDC. The ADHD-C group also demonstrated increased SC-FC coupling in the DMN compared to the ADHD-I group. The correlation between SC-FC coupling and hyperactivity scores was negative in the ADHD-I, but not in the ADHD-C group. The current study suggests that ADHD-C and ADHD-I may differ with respect to their underlying neuronal connectivity and that the added dimensionality of hyperactivity may not explain this distinction.

## 1. Introduction

Attention deficit hyperactivity disorder (ADHD) remains one of the most prevalent neurobehavioral disorders of childhood and adolescence, affecting social, emotional, and academic functioning. The DSM-V [1] segregates children with ADHD into defined subtypes based on the relative presentation of inattentive and hyperactive core symptoms (high inattention/low hyperactivity, ADHD-I; high hyperactivity/low inattention, ADHD-H; and, high inattention/high hyperactivity, ADHD-C). After early childhood, the prevalence of a purely ADHD-H subtype becomes less common, thus the presence of significant hyperactivity-impulsivity differentiates between the ADHD-I and ADHD-C subtypes during the later years and into adulthood [2, 3]. Although some fluidity between subtypes exists, separating the predominantly inattentive subtype from the combined group remains relevant with respect to risk for co-morbid diseases [4, 5], genetic associations [6–9], medication efficacy [10], and clinical course [see 11 for review]. The explanatory power of the subtype delegation suggests that examining the neural underpinnings of the distinction between ADHD-I and ADHD-C could help inform our understanding of the pathophysiology of this disorder, guide strategies for treatment, and help predict long term outcomes.

Neuroimaging findings in ADHD using diffusion tensor imaging (DTI) or resting-state functional MRI (rsfMRI) support the concept that a dysfunction in neurocircuitry may underlie this disorder and that differences in neural connectivity could distinguish between subtypes [see 12 for review]. Work on intrinsic networks have singled out the subnetworks associated with the behavioral, cognitive, and emotional symptoms related to ADHD. In particular, interest focused on the default mode network (DMN) in ADHD for its role in self-referential thought, non-goal directed activity, and “mind wandering” [13–16]. Based on the finding that inadequate suppression of the posterior DMN diminished performance on cognitive tasks [17], Sonuga-Barke and Castellanos [18] postulated the interference hypothesis of ADHD, suggesting that atypical functioning of the DMN may help explain the attentional deficits so prevalent in ADHD. Findings of reduced connectivity within the DMN [19], and diminished anti-correlations of the DMN with task specific networks both at rest [20–22] and during tasks in ADHD [23, 24] support this hypothesis, but many of the studies have not differentiated between subtype. Work on other intrinsic networks and their possible contribution to ADHD has focused on the executive control network (ECN) [25–28], and the salience network (SAN) [26, 29, 30], two networks associated with ADHD-related symptomatology. The ECN (also known as the lateral frontoparietal network), with anchoring nodes in the dorsolateral prefrontal cortex and the anterior inferior parietal lobule, orchestrates goal directed behavior, working memory and sustained attention [31]. The SAN encompasses nodes in the anterior insula and the dorsal cingulate cortex, with connections to subcortical and limbic structures. The SAN contributes to attentional switching and reward processing [32, 33] and may mediate the shifting from internal reflections associated with the DMN to the external orientation of the ECN [31]. A recent study demonstrated that, compared to typically developing children and children with ADHD-I, those with ADHD-C exhibit altered connectivity between the ECN and SAN [34]. We intended to build on previous network studies in ADHD by incorporating subtype analysis and focusing on three specific subnetworks (DMN, ECN, and SAN) involved in the core symptom domains of ADHD.

Although a number of studies have examined structural circuitry with DTI [35–39] and functional connectivity with rsfMRI [22, 25, 39–41], few have attempted to understand the differences between subtypes or have tried to integrate these separate modalities into a comparative analysis of network architecture. One study that applied non-negative matrix factorization on the data from the ADHD-200 competition, demonstrated that structural and functional graph theory features of the DMN clusters with the ADHD-I diagnosis [35].

According to the brain network theory, the structural network (white matter tracts) constrains the functional brain network (correlated functional activity), but functional connectivity also deviates from the observed structural architecture [42, 43]. In general, structural connectivity in the brain is highly associated with functional connectivity [43–45]. In the brain network topology, structural hubs nodes [44, 46] tend to play central roles in functional networks [47–49]. Despite a substantial correlation between structural and functional connectivity, functional connectivity is not fully constrained by the structural connectivity. No more than 50% of the functional connectivity measured at a macroscopic scale corresponds to structural architecture in a strictly linear manner [50]. Strong function connections exist for edges without direct structural connections [43] and vice versa [45, 51]. The structural connectivity and function connectivity are not uniformly related but gradually decouple according to the macroscale gradient [52].

The degree of coupling between the structural and functional networks (SC-FC) differs according to the brain state or pathology. SC-FC coupling can help characterize brain disorders such as schizophrenia [53], epilepsy [54], cerebral palsy [55], cognitive impairment [56], and bipolar disorder [57], and changes during typical development [58]. Despite the increasing use of SC-FC coupling in brain research, SC-FC coupling in ADHD subtypes remains unknown.

In this study, we explored the SC-FC coupling in the DMN, ECN, and SAN, intrinsic networks in the brain, using a database (N = 201, 75 children with ADHD with inattentive symptoms, 70 children with ADHD with combined symptoms, and 56 healthy children) from the Healthy Brain Network (HBN) database [59]. We primarily focused on network topologies for ADHD subtypes by exploring the structural and functional network properties and SC-FC couplings. This research reveals how structural and functional networks manifest differently both between ADHD subtypes, and between children with ADHD and typically developed children (TDC) in brain subnetworks relevant to ADHD.

## 2. Methods

### 2.1. Subjects

We used subject data from the Healthy Brain Network (HBN) database [59] for this analysis. The dataset, acquired from two medical centers, included total of 201 subjects: 70 children with ADHD-C (male = 56, female = 14, aged from 5.0 to 16.7 years, mean = 10.0, standard deviation (SD) = 2.4), 75 children ADHD-I (male = 54, female = 21, aged from 6.4 to 17.0 years, mean = 11.5, SD = 2.9) and 56 TDC (male = 20, female = 36, aged from 6.2 to 16.5 years, mean = 10.2. SD = 2.6) according to the primary diagnosis. There were significant group differences in age (F(1,2) = 6.14, p = 0.0026) and sex (F(1,2) = 16.66, p = 2.1×10^−7^). No significant differences in Wechsler Intelligence Scale for Children [WISC, 60] was found among groups (F(1,2) = 2.20, p = 0.1137). We received permission to access the Child mind institute biobank to use the public data, and it does not need any consent from the individuals for the current study.

Table 1 summarizes the demographics of the children’s data used in the current study.

**Table 1:** Demographics and clinical characteristics of ADHD and typically developing children.

		ADHD-C (N = 70)	ADHD-I (N = 75)	TDC (N = 56)	Difference between ADHD-C and ADHD-I, p-value	Difference among three groups, p-value (F-value)
Age, Years		10.0 ± 2.4	11.5 ± 2.9	10.2 ± 2.6	0.0012	0.0026 (6.14)
Sex	Male	56 (80.0%)	54 (72.0%)	20 (35.7%)	1.3×10^−7^	2.1×10^−7^ (16.66)
	Female	14 (20.0%)	21 (28.0%)	36 (64.3%)		
IQ profiles						
Full-scale		75.8 ± 41.3	84.6 ± 36.2	66.3 ± 53.8	0.1768	0.1137 (2.20)
Verbal Comprehension Index (VCI)		39.8 ± 35.0	47.1 ± 34.8	43.1 ± 38.8	0.2143	0.4899 (0.72)
Visual Spatial Index (VSI)		35.2 ± 31.9	44.4 ± 30.8	35.6 ± 36.2	0.0777	0.2028 (1.61)
Fluid Reasoning Index (FRI)		36.4± 34.5	43.1± 30.7	35.8 ± 35.5	0.2214	0.5216 (0.65)
Working Memory Index (WMI)		35.1± 33.5	33.0± 28.4	34.7± 34.4	0.6870	0.8722 (0.14)
Processing Speed Index (PSI)		26.1± 28.2	29.8 ± 27.2	37.5± 38.1	0.4282	0.1647 (1.82)
SWAN scores						
Hyperactivity average		1.2 ± 0.8	0.2 ± 1.1	−0.9 ± 1.1	2.9×10^−9^	3.6×10^−9^ (53.0)
Inattention average		1.4 ± 0.8	1.2 ±0.9	−0.8 ± 1.1	0.0674	2.9×10^−25^ (76.2)
Psychiatric comorbidity N(%)						
Autism Spectrum Disorder		6 (8.6)	9 (12.0)	-	-	-
Specific Learning Disorder		8 (11.4)	5 (6.7)	-	-	-
Communication Disorder		3 (4.3)	3 (4.0)	-	-	-
Motor Disorder		1 (1.4)	2 (2.7)	-	-	-
Intellectual Disability		1 (1.4)	1 (1.3)	-	-	-

ADHD: attention-deficit/hyperactivity disorder; ADHD-C: ADHD-Combined Type; ADHD-I: ADHD-Inattentive Type; TDC: Typically Developing Children; SWAN: the Strength and Weaknesses of ADHD-symptoms and Normal-behavior.

### 2.2. Data acquisition and processing

We analyzed 201 subjects’ diffusion MRI, resting-state fMRI, and T1-weighted MRI data selected from the available releases (1 and 2) of the HBN database [59] according to the following set of criteria. We sorted subjects with both diffusion imaging data and resting state functional data. The two experienced researchers in neuroimaging rated the image quality into three levels (good, moderate, and poor) according to fMRI inhomogeneity, the field of view outside of the brain, discontinuous slice intensities, severe motion artifacts, and spatial normalization problem. Images with good levels were used in the current study. Comparison of group differences in motion artifacts using framewise displacement [61], revealed a tendency toward, but no group difference (F(1,2) = 2.89, p = 0.058; TDC = 11.3 ± 1.2 (mean, std), ADHD-I = 12.1 ± 1.0, ADHD-C = 16.8 ± 1.6). A detailed description of MRI parameters can be found in Alexander et al. [59].

### 2.3. Construction of structural connectivity network

We constructed a structural connectivity network (SC) for each participant with 90 network nodes from cortical and subcortical regions defined according to the automated labeling (AAL) map [62]. The diffusion data obtained from the HBN database consisted of diffusion kurtosis imaging with multiple *b*-values (b = 0, 1000, 2000 s/mm^2^). Following the procedure for multiple b-value diffusion imaging data analysis [63], we used a conventional pipeline for probabilistic tractography from FMRIB’s diffusion toolbox (https://www.fmrib.ox.ac.uk/fsl) in the individual’s diffusion MRI space [64]. First, individual T1-weighted brain images were parcellated into 90 AAL regions by applying a nonlinear transformation of the AAL map in the template space using the DARTEL toolbox in SPM12 [65]. We then transformed the parcellated 90 regions into the diffusion MRI space by applying the transformation function of co-registration between the T1-weighted image to each individual’s non-diffusion weighted MRI image.

To create the structural networks, we performed “BedpostX” (Bayesian estimation of diffusion parameters obtained using sampling techniques) that implements Markov Chain Monte Carlo sampling to test propagation directions according to probabilistic orientation distribution at each voxel [64]. To estimate probability fiber density between AAL regions, we conducted “ProbtrackX” (probabilistic tracking with crossing fibers) with the following parameters: 5,000 samples for each voxel, 0.2 curvature threshold, 0.5 mm step length, and 2,000 steps per sample. Structural connectivity matrices were constructed by calculating the number of fibers (probabilistic streamlines) passing through the 90 brain regions. The probabilistic fiber tracking for 201 subjects was accelerated using Graphics Processing Units (GPUs, NVIDIA GeForce GTX 1080 Ti) [66].

### 2.4. Construction of functional connectivity network

To construct a functional connectivity network (FC), we conducted standard preprocessing of the fMRI data using SPM12 (http://www.fil.ion.ucl.ac.uk/spm, Wellcome Trust Centre for Neuroimaging, London, UK) [67] and an inhouse MATLAB (Mathworks, inc.) toolbox, MNeT (multimodal network analysis tool, http://neuroimage.yonsei.ac.kr/mnet/), which offers graph analytical functions for fMRI and DTI. All fMRI data underwent slice scan time correction, head motion correction, co-registration of T1-weighted images to the first EPI, and spatial normalization for converting the functional EPI into MNI template space using nonlinear transformation. The fMRI time-series for each of the 90 AAL regions used in the structural analysis was obtained from the normalized fMRI data by applying principal component analysis to all the voxel time series from each region to extract its representative temporal activity (eigenvariate of the first principal mode). We then regressed out six rigid motion parameters and their derivatives, three principal components from the white matter and the cerebrospinal fluid masks, linear and quadratic regressors. A high-pass filter with a cutoff frequency of 0.009Hz was applied. Functional connectivity matrices were constructed by calculating Fisher’s z-transformation of the correlation coefficients between every pair of the 90 brain regions.

### 2.5. Subnetwork analysis

**Fig 1:**
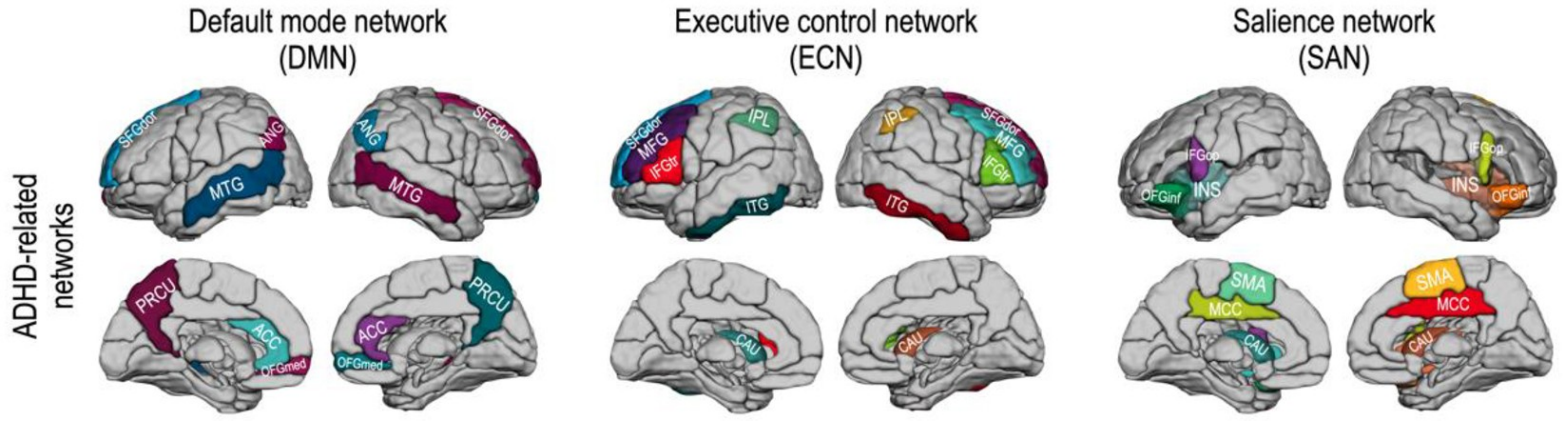
ADHD-related brain networks for subnetwork analysis—The default mode network (12 regions), executive control network (12 regions), and salience network (12 regions). Abbreviations: ANG, angular gyrus; PRCU, precuneus; ACC, anterior cingulate cortex; MCC, middle cingulate cortex; OFGmed, orbitofrontal cortex (medial part); MTG, middle temporal gyrus; SFGdor, superior frontal gyrus (dorsal part); MFG, middle frontal gyrus; IFGop, inferior frontal gyrus (pars orbitalis); IFGtr, inferior frontal gyrus (triangular part); IPL, inferior parietal lobe; CAU, caudate; INS, insular; MTG, middle temporal gyrus; ITG, inferior temporal gyrus; SMA, supplementary motor area.

To investigate the topological characteristics of ADHD-related brain subnetworks, we evaluated the structural and functional features of the DMN, ECN, and SAN. According to previous studies [68–70], we chose 12 brain regions of the AAL map for the DMN—the bilateral superior frontal gyrus (medial), middle temporal gyrus, superior frontal gyrus (dorsolateral), anterior cingulate cortex, precuneus, and angular gyrus. According to [71], we defined the ECN with 12 brain regions of the AAL map—the bilateral superior frontal gyrus (dorsolateral), middle frontal gyrus, inferior frontal gyrus (triangular part), inferior parietal gyrus, caudate, and inferior temporal gyrus. We also defined the SAN with twelve regions [71]—the middle cingulate cortex, supplementary motor area, inferior frontal gyrus (pars orbitalis), superior frontal gyrus (medial), insular cortex, and caudate bilaterally. These subnetworks were subparts of the whole brain network of 90 brain regions defined by the AAL map. Fig 1 displays ADHD-related brain subnetworks evaluated in this study.

### 2.6. Subnetwork properties and SC-FC couplings

As the structure-function coupling does not provide detailed information on whether the functional network deviates from the structural network in a positive (compensatory) or negative (as a result of pathology) mode relative to the symptom, we used global efficiency as an interpretive clue in terms of information transfer efficiency [55]. For each subnetwork, we calculated global efficiency of the structural and functional connectivity using the BCT toolbox [72]. For a network *G* with the total number of nodes *N*, global efficiency *E*_*glob*_ is defined as the average of all node efficiencies:

$$E_{glob}=\frac{1}{N\left(N-1\right)}\sum_{i\neq j\in G}\frac{1}{d\left(i,j\right)},$$

where *d* is the length of the shortest path between nodes *i* and *j*.

To evaluate global efficiency, we constructed the network by thresholding the connectivity matrices: structural networks were constructed with one or more fiber counts while functional networks were thresholded with a positive value corresponding to a FDR (false discovery rate) < 0.05.

For the metric of SC-FC coupling, we followed [58, 73] and calculated the Spearman correlation between the log-transformed structural network elements (numbers of fibers) and their functional connectivity (correlation coefficient of rsfMRI) among brain regions within the DMN, ECN, SAN, respectively, and for the whole brain for each subject. The log-transformation of the fiber counts was used to approximate a normal distribution [55, 74, 75]. In practice, the structural and functional connectivity matrices were vectorized (upper triangular), and then the SC-FC coupling was calculated using a Spearman correlation coefficient between them.

To reduce effects derived from the two different data acquisition sites, we harmonized the network property and SC-FC couplings using a ComBat model [76–78] that estimates site-specific and biological covariates in the Bayesian framework. For the ComBat model, subnetwork properties and couplings were modeled with data acquisition sites, age, and sex as the covariates of interest. We used a MATLAB code for ComBat analysis available from (https://github.com/Jfortin1/ComBatHarmonization).

### 2.7. Statistical analysis of subnetwork properties and SC-FC couplings

To evaluate group differences in the multi-site harmonized structural and functional connectivity features and the SC-FC coupling of the global network, we employed a one-way analysis of covariance (ANCOVA), with age and sex as covariates. For the functional connectivity analysis, we used FDR < 0.05 as the criteria for statistical significance.

In order to add a behavioral dimension, we conducted correlation analyses of the individual subject scores on both the Strength and Weaknesses of ADHD-symptoms and Normal-behavior (SWAN) battery with SC-FC coupling. First, we conducted a correlation analysis between hyperactivity and inattention scores to test whether a relationship between these scores manifests in either of the two subtypes. These two scores differentiate the two subtypes and construct two different domains. Second, we conducted a correlation analysis between the hyperactivity scores and SC-FC couplings and between the inattention scores and SC-FC couplings in the DMN to assess subtype differences.

## 3. Results

### 3.1. Connectivity differences in structural and functional networks

**Fig 2:**
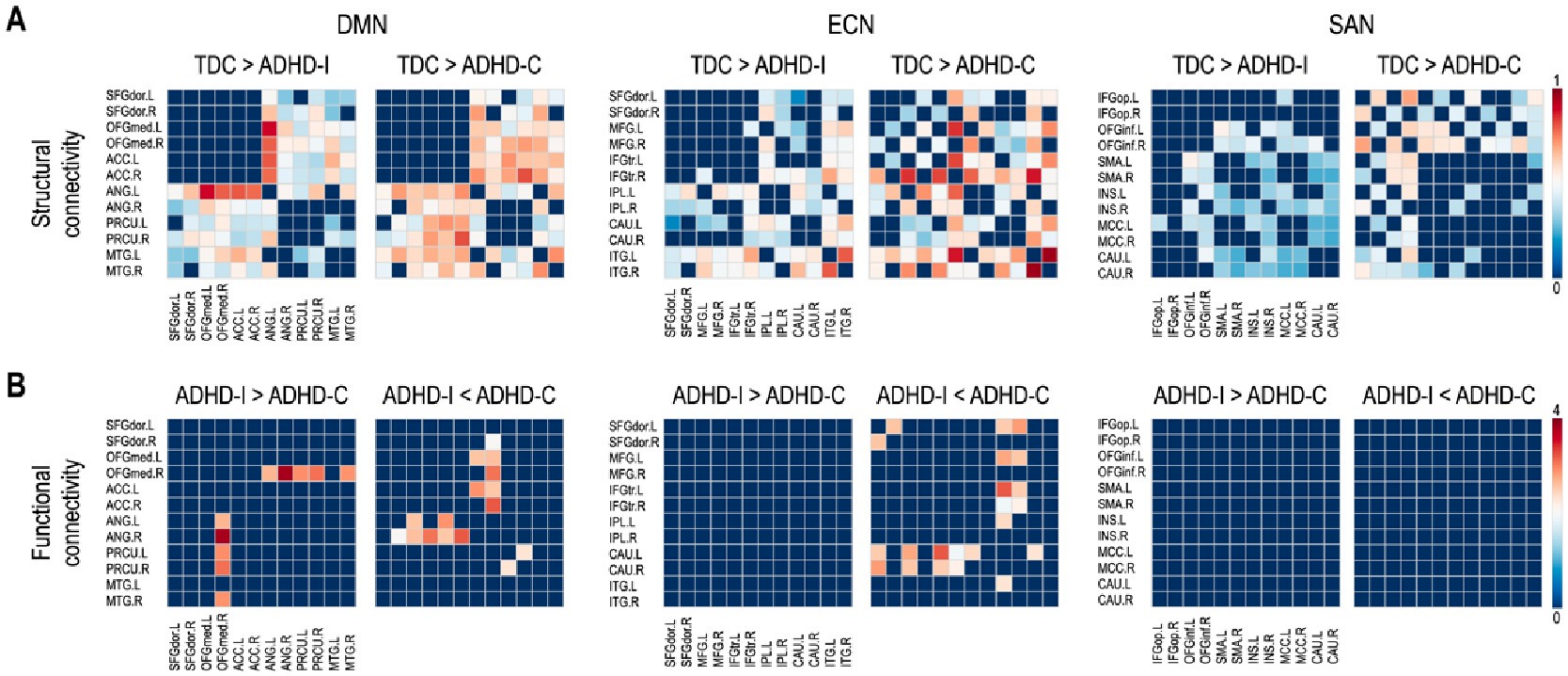
(A) Statistical group differences in structural connectivity between regions of the subnetworks (B) Because functional connectivity measures did not distinguish either ADHD subtype from TDC, only the difference in functional connectivity between the ADHD-I and ADHD-C subtypes are displayed (FDR < 0.05).

Fig 2 summarizes statistical group differences in connectivity between the edges (for structural connectivity) and the nodes (for functional connectivity) in each subnetwork. Two-sample t-tests demonstrated significantly lower connectivity across a wide range of structural regions in contrast to the lack of functional connectivity differences in ADHD-I and ADHD-C subjects compared to TDC.

The network analysis results of the whole brain network are presented in S1 Table. In the whole brain analysis, neither the functional efficiency nor the SC-FC couplings significantly differed between children with ADHD and TDC. However, global efficiency in the whole brain structural network was reduced in ADHD children compared to TDC, suggesting that architecturally, children with ADHD have less integrated anatomical connections over the entire brain (S1 Table). Table 2 summarizes the structural and functional efficiencies of the DMN, ECN, and SAN subnetworks between groups.

**Table 2:** Global network efficiency of structural connectivity (SC) and functional connectivity (FC), and the SC-FC couplings in ADHD-related subnetworks.

Global property & Coupling	Mean (Standard error of the mean)			P-value of ANCOVA (F-value)	P-values adjusted for multiple comparison across groups		
	TDC	ADHD-I	ADHD-C	TDC vs. ADHD-I vs. ADHD-C	TDC vs. ADHD-I	TDC vs. ADHD-C	ADHD-I vs. ADHD-C
**Default mode network**							
Global structural efficiency (SC)	0.6358 (0.0073)	0.5991 (0.0073)	0.5869 (0.0099)	*0.0009 (7.28)	*0.0032	*0.0013	0.9309
Global functional efficiency (FC)	0.5590 (0.0123)	0.5702 (0.0115)	0.5414 (0.0112)	0.5474 (0.60)	0.9999	0.6380	0.5868
SC-FC coupling	0.2691 (0.0221)	0.2668 (0.0201)	0.3675 (0.0198)	*0.0166 (4.18)	0.9986	0.0515	*0.0237
**Executive control network**							
Global structural efficiency (SC)	0.6637 (0.0074)	0.6385 (0.0070)	0.6243 (0.0086)	*0.0061 (5.24)	*0.0333	*0.0051	0.7294
Global functional efficiency (FC)	0.4871 (0.0117)	0.4917 (0.0095)	0.4955 (0.0096)	0.2364 (1.45)	0.9835	0.2983	0.3158
SC–FC coupling	0.0763 (0.0230)	0.0972 (0.0209)	0.1763 (0.0211)	*0.0084 (4.90)	0.2639	*0.0052	0.1829
**Salience network**							
Global structural efficiency (SC)	0.6859 (0.0052)	0.6609 (0.0063)	0.6528 (0.0081)	*0.0015 (6.70)	*0.006	*0.0019	0.8904
Global functional efficiency (FC)	0.4733 (0.0129)	0.4798 (0.0117)	0.4818 (0.0111)	0.6510 (0.43)	0.8383	0.6233	0.9101
SC–FC coupling	0.0735 (0.0203)	0.0572 (0.0130)	0.1307 (0.0174)	0.063 (2.80)	0.8772	0.170	0.0686

*Significant group differences (p<0.05) after ANCOVA.

**Fig 3:**
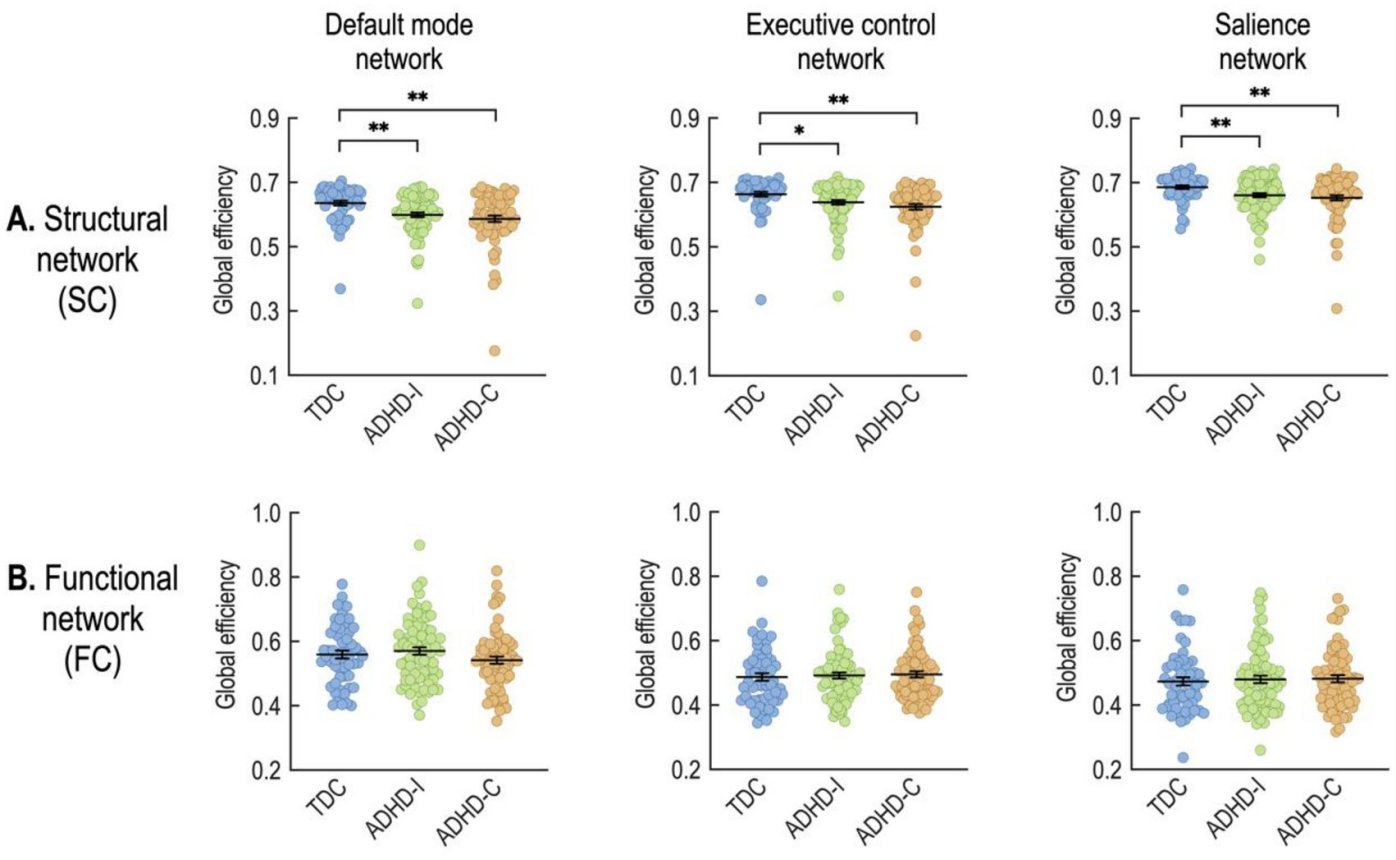
Group comparisons of global structural efficiency (A) and global functional efficiency (B) in the default mode, executive control, and salience networks. *p<0.05, **p<0.01 (two-sample t-tests).

The analysis of covariance (ANCOVA) with age and sex as covariates revealed significant group differences in global efficiency of the structural connectivity within the DMN (F(1,2) = 7.28, p = 0.0009), ECN (F(1,2) = 5.24, p = 0.0061), and SAN (F(1,2) = 6.70, p = 0.0015). As shown in Fig 3, compared to controls, both ADHD-C and ADHD-I groups demonstrated significantly diminished efficiency of the structural connectivity within all three subnetworks (DMN, ECN, and SAN). Whereas, no statistical differences in the global efficiency of the functional connectivity within the DMN, ECN, or SAN emerged between ADHD subtypes and TDC (Table 2).

### 3.2. Structure-function coupling differences

**Fig 4:**
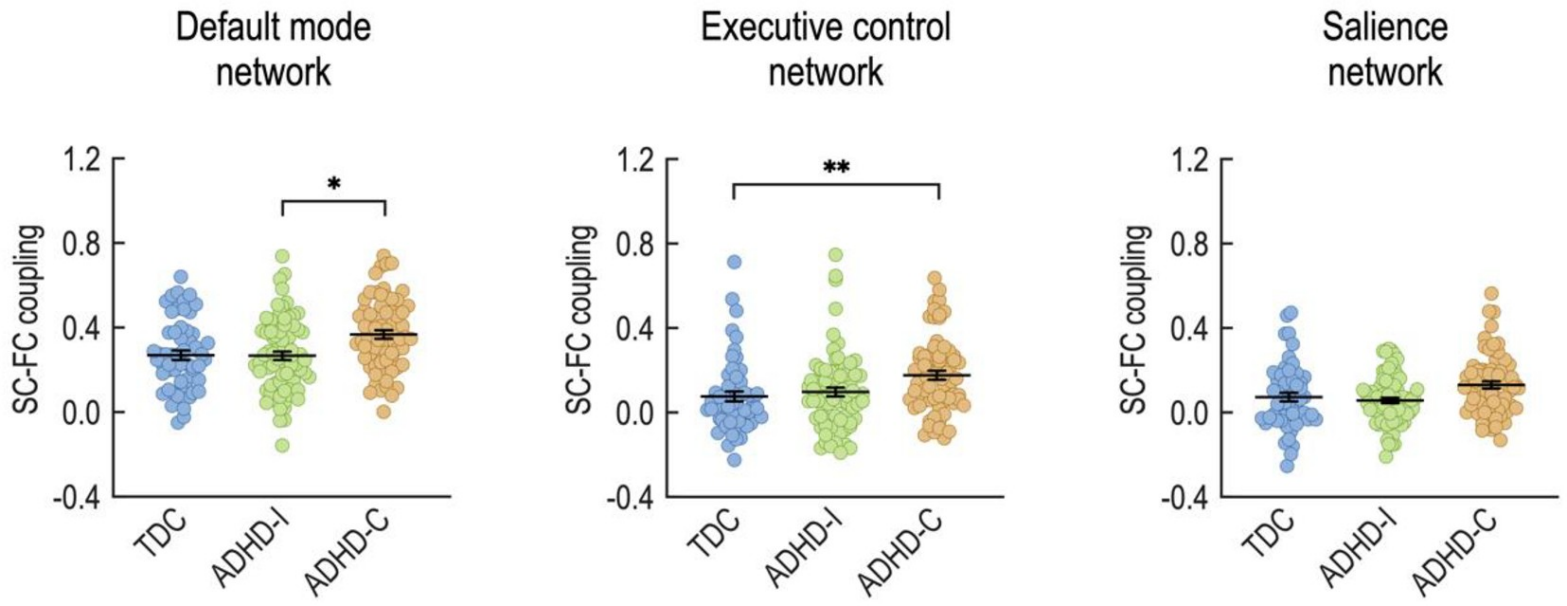
Structure-function coupling results for the ADHD-related subnetworks. *p<0.05. **p<0.01 (two-sample t-tests).

Fig 4 depicts the SC-FC coupling results for the three subnetworks. The SC-FC coupling analysis revealed significant group differences in the DMN (F(1,2) = 4.18, p = 0.0166; one-way ANCOVA with age and sex as covariates), ECN (F(1,2) = 4.90, p = 0.0084), and a tendency in the SAN (F(1,2) = 2.80, p = 0.063). Specifically, the ADHD-C group exhibited significantly higher SC-FC coupling than the ADHD-I group in the DMN (two-sample t-tests, ADHD-C = 0.37 (mean) ± 0.02 (standard error of the mean), ADHD-I = 0.27 ± 0.02, p = 0.0237). ADHD-C also displayed significantly higher SC-FC couplings in the ECN (ADHD-C = 0.18 ± 0.02, TDC = 0.08 ± 0.02, p = 0.0052) compared to TDC.

### 3.3. Relationship between ADHD-related behavioral scores and structure-function couplings

**Fig 5:**
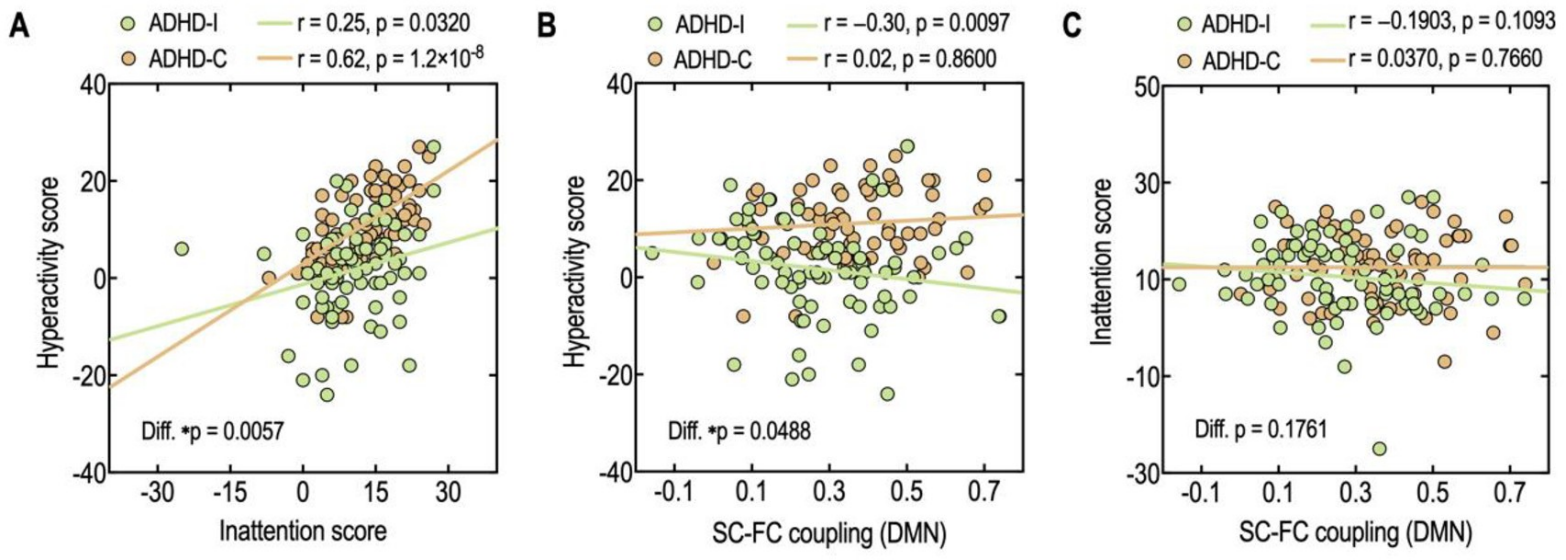
Relationship between inattention and hyperactivity (A), between SC-FC coupling in the DMN and hyperactivity score (B) between SC-FC coupling in the DMN and inattention score (C) in ADHD-I and ADHD-C. Diff: statistical significance of the two different correlation coefficients.

The correlation analysis showed that inattention scores positively correlate with hyperactivity scores in both ADHD-I (r = 0.25, p = 0.0320) and ADHD-C (r = 0.62, p = 1.2 × 10^−8^) (Fig 5A). A statistical comparison of the correlation coefficients [79] reveals a stronger relationship between inattention and hyperactivity in the combined type compared to the inattentive subtypes (p = 0.0057). When we removed the outliers in ADHD-I (7 ADHD-I children), the correlation coefficients were r = 0.28 (p = 0.0244) for ADHD-I, and the relationship between inattention and hyperactivity is still significantly different across subtypes (p = 0.0112).

The SC-FC coupling in the DMN of ADHD-I demonstrated a significant negative correlation with the hyperactivity score (r = −0.30, p = 0.0097) (Fig 5B); whereas, the SC-FC coupling in the DMN of ADHD-C did not correlate with the hyperactivity score (r = 0.02, p = 0.8600). The relationship between SC-FC coupling and hyperactivity scores differed significantly between the two subtypes. The ADHD-I group exhibited a significantly different correlation between SC-FC coupling in the DMN and hyperactivity than ADHD-C (p = 0.0488). No significant correlations were found between the SC-FC coupling and the inattention score in either ADHD-I (r = −0.1903, p = 0.1093) or ADHD-C (r = 0.0370, p = 0.7660) (Fig 5C).

**Fig 6:**
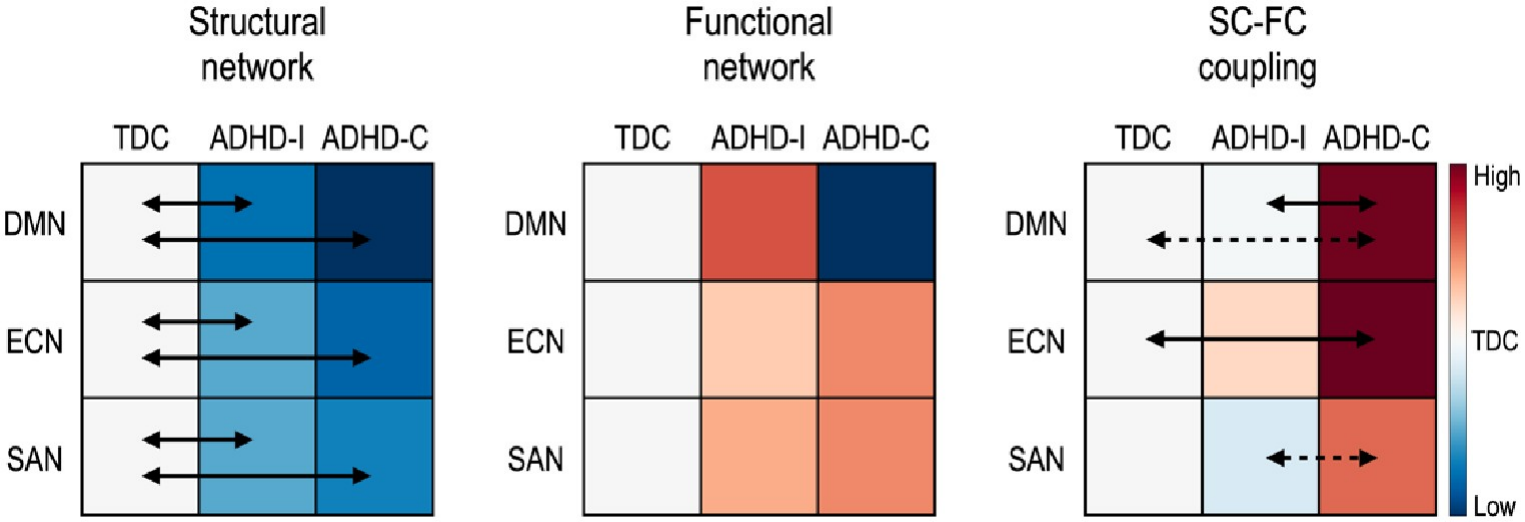
Summary of the global efficiency of both structural connectivity (SC) and functional connectivity (FC), and the SC-FC couplings in ADHD-related subnetworks. The solid arrow indicates significant group differences and the dotted arrow indicates a tendency of differences (if not significant) between groups. The colors indicate the group means of each feature relative to the TDC. Red indicates higher mean value while blue indicates lower mean value than TDC. DMN: default mode network, ECN: executive control network, and SAN: salience network.

Fig 6 graphically summarizes the current results.

## 4. Discussion

In the brain subnetworks relevant to ADHD, an analysis of both structural efficiency and SC-FC coupling reveals differences between ADHD-I, ADHD-C, and TDC.

The structural properties differ more than the functional properties of networks between children with ADHD and TDC. Only structural connectivity (see Fig 2) and the efficiency of the structural network were found to be lower in children with ADHD compared to TDC (Fig 3). The enhanced sensitivity of structural networks rather than functional networks to neuropsychiatric symptoms has been reported in other developmental brain diseases, such as cerebral palsy [55].

We found lower global structural efficiency in the whole-brain network of children with ADHD (S1 Table) compared to TDC. Global structural efficiency measures the capacity for the network to transmit information across the entire expanse of its geometry (an approximation of integration) (S1 Table). This finding corroborates the previous work of Cao, Shu [36] demonstrating that the underlying structural architecture of the brain in ADHD may not support the transfer of information in the same manner as TDC. Although the group difference between ADHD and TDC was significant, the structural network efficiency did not differ between the ADHD-I and the ADHD-C in the whole-brain network analysis. No significant SC-FC coupling differences exist between groups in the whole-brain network (see S1 Table).

To explore characteristics specific to ADHD subtypes, we focused on ADHD-related subnetworks, the DMN, ECN, and SAN, which other groups have used to identify impairments in various brain diseases [53, 55, 73]. In an attempt to understand the pathophysiology of ADHD, many previous studies have analyzed the DMN [20, 22, 28, 35, 80, 81], ECN [25–28], and SAN [26, 29, 30]. This study adds to previous work by integrating structural, functional, and structure/function coupling and by refining the comparisons to include ADHD subtypes.

Similar to our whole-brain analysis, we found reduced global efficiency in the structural component of the DMN, ECN, and SAN in children with ADHD (more reduced in the ADHD-C group) relative to that in the TDC group (Table 2). Again, no significant differences in functional network efficiency emerged between the groups. Children with ADHD-C have significantly increased SC-FC coupling within the ECN and tend to have higher SC-FC coupling within the DMN (p = 0.0515) than TDC. The SC-FC coupling within the DMN, however, did differ between ADHD subtypes. The ADHD-C drove this finding by exhibiting enhanced SC-FC coupling relative to the other groups. Compared to separate analyses of SC and FC network properties, SC-FC coupling directly associates SC and FC within each individual. The finding that SC-FC coupling is stronger in ADHD-C compared to ADHD-I and TDC (Table 2) suggests that the functional organization more closely adheres to the structural networks in ADHD-C compared to ADHD-I.

The fundamental question of how flexible functions emerge from the structural network has prompted interest in the relationship between structural and functional connectivity [42, 43]. The structural network constrains the functional brain network [43–45] but not wholly [50]. Strong functional connections exist between brain regions without direct structural connections [43] and vice versa [45, 51]. Multiple synaptic connections or shared inputs could explain how functional correlations might diverge from the structural network [50]. The degree of divergence (or convergence) between the structural and functional networks has been measured using the correlation-coefficient based SC-FC coupling. Several studies have employed SC-FC coupling to characterize brain disorders such as schizophrenia [53], epilepsy [54], cerebral palsy [55], cognitive impairment [56], and bipolar disorder [57].

In these studies, high SC-FC coupling (convergent to the structural network) has often been interpreted as a marker of rigidity or inflexibility at the cognitive or behavioral level. However, the biological mechanism of SC-FC coupling is not fully understood. Multifactorial explanations for a higher order relationship between structure and function include such possibilities as polysynaptic connections, anti-correlations via connections to an inhibitory input, and differences in neuromodulation [50]. Jang, Knight [82] demonstrate that individuality or inter-individual differences during movie viewing are more apparent at the heterogeneous time courses of functional connectivity uncoupled from structural connectivity (e.g., polysynaptic connectivity). The negative correlations between specific subnetworks, for example, between the DMN and the frontoparietal network (similar to the ECN), may reduce SC-FC coupling [83].

The strong coupling in the DMN in children with ADHD-C compared to children with ADHD-I suggests that the DMN in the ADHD-C group may operate with fewer indirect polysynaptic connections. Whereas, at the symptomatic level, the negative correlation between hyperactivity and SC-FC coupling indicates that this symptom domain may contribute to cognitive control in the ADHD-I group. This aspect of executive functioning includes both the ability to stop one type of behavior and the capacity to engage with a different interest or initiate an alternative behavior. Incorporating measures of both cognitive flexibility and cognitive control into future studies of ADHD subtypes and SC-FC coupling could help evaluate the significance of this finding. The SC-FC coupling measure (correlation coefficient) indicates how much the functional network converges to the “baseline” structural network. Thus, the rigidity or inflexibility (the degree of convergence) should be interpreted concerning the “inefficient” structural network in ADHD, particularly in the ADHD-C subtype.

In the ADHD-C subtype, the interactions between brain regions in the DMN appear to be strongly constrained by an inefficient structural network topology. Considering that the DMN is involved in self-reflection, mind wandering, and internally oriented processes [13–16, 84], the inefficient structural topology for paying attention to the inward self might lead to the externalization of outward hyperactivity. However, SC-FC coupling in the DMN in ADHD-C cannot be explained by inefficient structural network topology alone because children with ADHD-I also have inefficient structural topology in the DMN (Fig 3). In contrast to children with ADHD-C, children with ADHD-I had SC-FC coupling in the DMN at a similar level to that in TDC.

Currently, the DSM-V [1] segregates ADHD into ADHD-C and ADHD-I in a dimensional paradigm and considers the combined type to include hyperactivity in addition to the inattentive dimension in ADHD-I. However, the current study suggests that ADHD-C and ADHD-I may have different biological bases that cannot be explained purely by the additive dimensionality of hyperactivity. Indeed, a differential relationship between hyperactivity and SC-FC coupling exists between ADHD-I and ADHD-C. The DMN SC-FC does not correlate with SWAN hyperactivity scores in ADHD-C but negatively correlates with ADHD-I (Fig 5B). Thus, ADHD-C might not be purely considered as ADHD-I plus hyperactivity. Inattentiveness and hyperactivity may have differential interactions in both groups (Fig 5A). In the SWAN scores, the relationship between hyperactivity scores and inattention scores was significant in both the ADHD-C and ADHD-I groups, but the degree of correlation was significantly higher in the ADHD-C group than in the ADHD-I group. In this respect, the independency assumption between inattentiveness and hyperactivity may not hold, although we acknowledge the limitation inherent in the survey format of SWAN. These speculations should be further explored in future studies.

Although we have primarily explained the DMN of children with ADHD-C in terms of functional inflexibility over an inefficient structural topology, the ECN and SAN exhibit comparable degrees of structural network inefficiency. The ADHD-C group displayed higher SC-FC coupling in the ECN compared to controls, but no significant difference in SC-FC coupling between ADHD subtypes emerged. Of note, the SC-FC couplings in the SAN and ECN are relatively weak compared to the coupling observed in the DMN. A portion of the children measure in the zero-coupling zone (zero correlation coefficients). In addition, some children display anti-correlated SC-FC coupling in the SAN and ECN. A negative functional connectivity transmitted over a structural edge may explain the anti-correlated coupling between the SC and FC subnetworks of these children. Thus, the ECN and SAN may not submit to the same explanation as the DMN.

We cannot generalize that the relationship between hyperactivity symptoms and the SC-FC coupling in the DMN extends to the other subnetworks. In addition, the inattentive symptoms in both ADHD-I and ADHD-C may not be attributable to dysfunction in a specific subnetwork but may relate to a combination of different subnetworks, for example, the ECN, SAN, and DMN, or to abnormal interactions between networks. These subnetworks may function differently in TDC, in children with ADHD-I, and in children with ADHD-C. This interpretation is consistent with the constellation of multiple dysfunctions witnessed in the DMN [12, 85], ECN [25–28], and SAN [26, 30, 36] reported in previous structural and functional neuroimaging studies of ADHD.

This study has several limitations. First, our control group of TDC does not reflect the gender imbalance found in the ADHD samples. A recent metanalysis of human connectome studies did not find significant gender differences [86], but this may not apply to children with ADHD. The current study should be interpreted in consideration of the male-gender bias (Table 1).

Second, the Healthy Brain Network (HBN) database incompletely documents the history of stimulant use and current medication status of the individual participants—Only 51 children over age 10 evaluated in the current study had a complete drug screening before the MRI scans. Prolonged stimulant use can enhance gray matter volume [87] and may improve white matter integrity [88]. Because treatment with stimulants is more common in children with ADHD-C than in ADHD-I, detailed knowledge of medication use could have affected our interpretation of the results.

Third, when the current study was conducted, the MRI and behavioral data of the release 1 and 2 were fully accessible. Thus, we consider the current results preliminary at this point and leave the analysis with more detailed parcellation maps and more data for a further study.

Fourth, graph analysis of the brain requires the nodes, or anatomical parcellations to cover the entire cortex. We have followed this stipulation, but in doing so, we may have obscured more fine-grained details about the connectivity between brain regions. In particular, the extraction of time courses from relatively large brain regions that may support varied functions could effectively have washed out group differences in functional connectivity. Conversely, we cannot rule out that the heterogeneous composition of a node used in this study may increase the detection power between groups considering context or state-dependent changes in the functional atlas even within an individual [89]. Since the AAL map used in the current study is not a functional atlas and thus the subsystems of the current study, i.e., DMN, ECN and SAN do not fully overlap with the functional connectivity-based atlases [e.g., 69, 90]. Thus, the subsystems of the current study may include heterogeneous compartments in terms of intrinsic functional connectivity within a parceled region of the AAL map and should be interpreted as such. We did not evaluate the SC-FC network coupling in diverse parcellation maps and data from all the datasets (release 9) of the HBN database for practical limitation of computational cost. For 90 nodes in the AAL map, it takes almost 2 hours to calculate an SC network of an individual using a GPU (NVIDIA GeForce GTX 1080Ti) (10 hours for Intel Zeon CPU), which makes it advantageous to calculate the whole 201 children’s data compared to a highly precise atlas with large nodes.

Fifth, the computations used to establish the functional connectivity of networks in this study do not incorporate deactivations. Because cognitive tasks and attention may rely on the DMN’s ability to effectively deactivate with respect to other networks, such as the ECN and the SAN, this analysis may overlook a crucial mechanism for the DMN’s role in ADHD. Further studies on the coupling between networks may be able to address this issue more successfully. Finally, the reliability of the functional connectivity of rsfMRI has been an issue [91]. Thus, we primarily focused on the SC-FC coupling as a type of multivariate analysis, which is advantageous in terms of reliability [92].

## 5. Conclusion

In summary, we investigated attention-related brain subnetworks in children with ADHD-I and ADHD-C using the relationship between structural and functional networks. We found SC-FC coupling differences between children with ADHD and TDC and between ADHD subtypes in the ADHD-related subnetworks (DMN, ECN, and SAN). Although SC-FC coupling in the DMN significantly differed between ADHD subtypes, the subnetworks seem to work together to manifest a different degree of inattentiveness and hyperactivity in the ADHD-I and ADHD-C groups. Given that structural differences between individuals with ADHD and controls has been demonstrated in children but not adults, further work on the SC-FC coupling should include longitudinal data [93]. These findings suggest that the structure-function network relationship may provide important neurobiological clues for understanding ADHD subtypes in children.

## Supporting information

S1 Table Global network properties of structural connectivity (SC) and functional connectivity (FC), and structure–function couplings in the whole-brain network. (DOCX)

S1 Data (ZIP)
