# Supplementary material for: Differential structure-function network coupling in the inattentive and combined types of attention deficit hyperactivity disorder
Source: PLoS One. 2021 Dec 1;16(12):e0260295. doi: 10.1371/journal.pone.0260295 (PMC8635373; doi:10.1371/journal.pone.0260295)
Supplement: S1 Table — (DOCX) [file pone.0260295.s001.docx]

**S1 Table.** Global network properties of structural connectivity (SC) and functional connectivity (FC), and structure–function couplings in the whole-brain network.

| Global property  &  Coupling | Mean  (Standard error of the mean) | | | P-value of ANCOVA  (F-value) | | P-value  for multiple comparison | | |
| --- | --- | --- | --- | --- | --- | --- | --- | --- |
|  | TDC | ADHD-I | ADHD-C | TDC  vs. ADHD-I  vs.  ADHD-C | TDC  vs. ADHD-I | | TDC  vs. ADHD-C | ADHD-I vs. ADHD-C |
| Global  efficiency (SC) | 0.6085  (0.0065) | 0.5739  (0.0059) | 0.5694  (0.0072) | *0.0006  (7.69) | *0.0011 | | *0.0019 | 0.9973 |
| Global  efficiency (FC) | 0.4018  (0.0091) | 0.4155  (0.0080) | 0.4078  (0.0075) | 0.4557  (0.79) | 0.4633 | | 0.9542 | 0.6243 |
| SC–FC coupling | 0.2052  (0.0081) | 0.2082  (0.0083) | 0.2160  (0.0088) | 0.8093  (0.21) | 0.9927 | | 0.8256 | 0.8588 |
